# Evaluation of Minimum Inhibitory Concentrations for 154 *Mycoplasma synoviae* isolates from Italy collected during 2012-2017

**DOI:** 10.1371/journal.pone.0224903

**Published:** 2019-11-07

**Authors:** Salvatore Catania, Marco Bottinelli, Alice Fincato, Michele Gastaldelli, Antonio Barberio, Federica Gobbo, Gaddo Vicenzoni

**Affiliations:** Istituto Zooprofilattico Sperimentale delle Venezie, viale Dell’Università 10, Legnaro (PD), Italy; Ross University School of Veterinary Medicine, SAINT KITTS AND NEVIS

## Abstract

*Mycoplasma synoviae* (MS) is a highly prevalent bacterial species in poultry causing disease and severe economic losses. Antibiotic treatment is one of the control strategies that can be applied to contain clinical outbreaks in MS-free flocks, especially because this bacterium can be transmitted *in ovo*. It becomes, then, very important for veterinarians to know the antibiotic susceptibility of the circulating strains in order to choose the most appropriate first-line antibiotic molecule as a proactive role in fighting antibiotic resistance. We evaluated the Minimum Inhibitory Concentrations (MICs) of enrofloxacin, oxytetracycline, doxycycline, erythromycin, tylosin, tilmicosin, spiramycin, tiamulin, florfenicol and lincomycin for MS isolates collected between 2012 and 2017 in Italy. A total of 154 MS isolates from different poultry commercial categories (broiler, layer, and turkey sectors) was tested using commercial MIC plates. All MS isolates showed very high MIC values of erythromycin (MIC90 ≥8 μg/mL) and enrofloxacin (MIC90 ≥16 μg/mL). MIC values of doxycycline and oxytetracycline obtained were superimposable to each other with only a one-fold dilution difference. Discrepancies between MIC values of tylosin and tilmicosin were observed. Interestingly, seven isolates showed very high MIC values of lincomycin and tilmicosin, but not all of them showed very high MIC values of tylosin. Most of the MS isolates showed low MIC values of spiramycin, but seven strains showed a MIC ≥16 μg/mL. In the observation period, the frequency of the different MIC classes varied dependently on the tested antibiotic. Interestingly, tilmicosin MICs clearly showed a time-dependent progressive shift towards high-concentration classes, indicative of an on-going selection process among MS isolates. Until standardized breakpoints become available to facilitate data interpretation, it will be fundamental to continue studying MIC value fluctuations in the meantime in order to create a significant database that would facilitate veterinarians in selecting the proper drug for treating this impactful *Mycoplasma*.

## Introduction

Several *Mycoplasma* species are considered significant pathogens for the poultry industry, among which *Mycoplasma gallisepticum* (MG) and *Mycoplasma synoviae* (MS) are considered the most relevant. These pathogens induce respiratory syndrome and articular disease and, as a consequence, severe economic losses especially in meat categories and in layers [1]. It has been reported that MS prevalence in layers can range from 30% to 95% [2]. The control of these mycoplasmas is mainly based on strict biosecurity measures, vaccination protocols (if available), and specific eradication programs in breeder flocks because of the evidence of vertical transmission [1].

Historically, a lot of attention was focused on MG, leaving to MS the role of a minor pathogen in some poultry categories; thus specific eradication programs were applied worldwide especially against MG in order to decrease its prevalence [1]. Despite all these efforts, MS prevalence remains high in the poultry industry suggesting there may be a lack of knowledge in their epidemiology or in the application of appropriate control measures. Recently, several European authors have been reporting outbreaks more frequently involving MS rather than MG, pointing out the real impact of MS on the poultry industry [2].

MS infection may cause synovitis in breeder chickens and turkeys resulting in reluctance to movement which negatively affects the growth performances and can lead to body lesions with consequent higher condemnation rate of the animals. MS infection is also associated with respiratory disease characterized by conjunctivitis, tracheitis and airsacculitis. When coinfection with other pathogens occurs, an even higher rate of production rejects at processing is observed [3]. In addition, since 2009 a new MS-related syndrome in layers characterized by the presence of eggshell alterations classified as “*Eggshell Apex Abnormalities*” (EAA) has been reported [4,5]. This disease has been observed worldwide and its economic impact is becoming more and more significant [4,5,6,7,8].

Because of the high MS infection prevalence in the poultry sectors in Europe [2], several control strategies have been proposed and applied to contain the clinical outbreaks and to guarantee the animal welfare; including the application of strict biosecurity measures, eradication programs involving depopulation of infected birds, vaccination or antibiotic treatments. Even though only few field studies on antimicrobial therapy are available in literature [9,10], it is well known that antibiotic treatment can only mitigate the clinical symptoms and it is not sufficient alone for the eradication of the infection in affected flocks [11]. This could be explained by the chronic behaviour of MS infection [3] and by the high presence of the microorganism in the environment [12]. Also, MS molecular mechanisms of acquired antibiotic resistance have been reported [13,14,15,16]. As a consequence, knowing the antibiotic susceptibility of the circulating strains becomes very important for better managing the drug therapy, avoiding then any selective pressure that may promote the development of antibiotic resistance. For this purpose the calculation of the Minimum Inhibitory Concentration (MIC) of drugs *via* agar dilution test, broth micro-dilution test or stripped-test are the common adopted techniques among laboratories.

Only few reports on MIC values for MS published in the past five years are available in literature despite the need of updated data [15,17,18,19,20,21,22,23,24,25]. Furthermore, there are differences in MIC values reported in recent works compared to past ones. Indeed, although most authors reported low MIC values for most licensed macrolides (except for erythromycin showing very high MIC values) [14,16,17,26,27,28], Lysnyansky and collaborators [16] have recently found some MS isolates with high MIC values for tylosin and tilmicosin. Doxycycline has been typically showing good activity against MS [20,21,22,23] but other authors observed decreased susceptibility or resistance to oxytetracycline in other Countries [18,27]. In addition, several authors have been reporting decreased susceptibility to fluoroquinolones worldwide [20,21,23,24] and elucidations on molecular acquired resistance to enrofloxacin in MS were proposed [13,15].

All things considered, the high prevalence of MS in the poultry sectors, the chronic behaviour of its infection (especially in long-cycle poultry production), the recurrence of the disease, and the eventuality of acquisition and spread of resistance to antimicrobials make it necessary to get recent MIC data on MS isolates causing disease. Accordingly, the aim of this study was to evaluate the MICs for Italian MS isolates recently collected in different commercial categories (broiler, layer, and turkey sectors).

## Material and methods

### *Mycoplasma synoviae* isolates

A total of 154 MS isolates was selected from the isolates collection of the Mycoplasma Unit of the Istituto Zooprofilattico Sperimentale delle Venezie on the base of the following criteria: year of isolation, avian species, poultry sector and genotype (see S1 Table). This selection was made with the intention of representing, as far as possible, the field epidemiological situation between 2012 and 2017. Therefore, a proportional number of isolates belonging to different poultry categories and originating from several Italian areas was included. Each isolate is derived from a single outbreak; if an outbreak occurred in a farm with different barns, only one isolate was included in the study in a 12-month time frame. Ninety-five out of 154 isolates (61.7%) belong to the broiler sector, 33 (21.4%) to the layer sector and 26 (16.9%) to the turkey sector. Thirteen isolates (8.5%) were collected in 2012, 25 (16.2%) in 2013, 32 (20.8%) in 2014, 35 (22.7%) in 2015, 35 (22.7%) in 2016 and 14 (9.1%) in 2017. The detailed data of MS isolates examined in this study are reported in Table 1.

**Table 1:** Number of isolates, year of isolation and poultry sector.

Year of isolation	Broiler	Layer	Turkey	Total
2012	3	10	-	13
2013	15	4	6	25
2014	16	6	10	32
2015	28	5	2	35
2016	25	4	6	35
2017	8	4	2	14
Total	95	33	26	154

### *In vitro* cultivation of the isolates

The 154 isolates derived from field or necropsy activities and were obtained by collecting tracheal swabs in clinically-affected birds or bird carcasses showing gross pathology findings related to MS infection. Tracheal swabs were rapidly inoculated in a transport medium or in a selective medium (Avian Mycoplasma Liquid Medium, Mycoplasma Experience®, Reigate, UK) and submitted to the laboratory for *Mycoplasma* spp. cultivation. The inoculated broths were incubated at 37±1°C under 5% CO_2_ conditions for at least 14 days. During this time the broths were checked daily and, when a change in colour (from orange to yellow) or turbidity was observed, they were inoculated onto an agar plate of Avian Mycoplasma Agar (Mycoplasma Experience^®^, Reigate, UK). If no change was seen after 14 days, a blind passage into a plate with solid medium of Mycoplasma Experience® was performed and plates were checked daily for the presence of any “fried-egg-like” colony. If no colony was observed during the following 7 days, samples were then considered negative.

### *Mycoplasma synoviae* identification and variable lipoprotein hemagglutining A gene (*vlhA)* genotyping

The *vlhA* genotyping was performed to exclude the presence of vaccine strains and to select the isolates with the final aim of representing the Italian prevalence during the study time. In order to perform *Mycoplasma* species identification, an aliquot of 300 μL from positive suspect broths was collected for DNA extraction. DNA was extracted using the Maxwell® 16 Blood DNA Purification Kit (Promega Italia Srl, Milano, IT). As a second step, 16S-rDNA PCR and Denaturing Gradient Gel Electrophoresis (DGGE) were performed on extracted DNA as described in the literature [29]. For the classification of the MS isolates, *vlh*A gene analysis [30] was carried out on each isolate. Briefly, once the PCR products were obtained, they were cleaned up using the Performa DTR Ultra 96-Well kit (Edge BioSystems, Gaithersburg, MD) and sequenced using BigDye Terminator v3.1 cycle sequencing kit (Applied Biosystems, Foster City, CA) in a 16-capillary ABI PRISM 3130xl Genetic Analyzer (Applied Biosystems, Foster City, CA). Sequence data were assembled and edited with SeqScape software v2.5 (Applied Biosystems). The codes of the different types of MS sequences followed the scheme described by Benčina et al. [31]. Isolates showing a *vlh*A profile identical to the MSH strain [32–34] were excluded.

### MIC test for *Mycoplasma synoviae*

The MIC procedure was carried out via broth microdilution technique following our internal procedure based on the guidelines of Hannan with slight modifications [19], and the standardized procedure, available for Human mycoplasmas [35]. As it is essential for MIC testing to use pure bacterial cultures, each MS isolate underwent three consecutive, *in vitro* passages, first in liquid and then in solid media, as described by Markey et al. [36] with slight modifications. At the third passage on liquid medium, the MS suspension was propagated in 10 mL of Mycoplasma Experience® (ME, Reigate, UK) without inhibitors. This bacterial stock solution in the exponential growth phase was divided in 5 sub-aliquots, each one of 1 mL, and stored at -80°C for at least 24 hours. On the following day, an aliquot was thawed and used for titration in 96-well microtitre plates for the calculation of the Unit Changing Colour (UCC/mL) as described in literature through the most probable number method [19,35,37]. Moreover, before performing the MIC test, each isolate was tested again by DGGE and for *vlh*A analysis as previously described in order to confirm the presence of only the selected MS isolate. When the titre was available, a new frozen sub-aliquot was thawed and diluted in liquid medium without inhibitors in order to achieve a standard final inoculum in the well of approximately 10^4^ UCC/mL. Commercial, custom-made 96-well microtitre plates containing lyophilized antibiotics (Merlin Diagnostik®, LOT ES-295-100 140919P95001) were used. The antimicrobial agents included in the MIC plate wells were enrofloxacin, oxytetracycline, doxycycline, erythromycin, tylosin, tilmicosin, spiramycin, tiamulin, florfenicol and lincomycin. The antibiotic concentration ranges, expressed in μg/mL, are reported in Table 2. On each plate, one well containing no antibiotics was inoculated with mere liquid medium and used as negative control.

**Table 2:** Antimicrobial susceptibilities patterns of the 154 *Mycoplasma synoviae* isolates tested.

Antibiotic	Dilution range (μg/mL)	Minimum MIC value (μg/mL)	Maximum MIC value (μg/mL)	MIC 50 (μg/mL)	MIC 90 (μg/mL)
Doxycycline	0.125–16	≤ 0.125	2	0.5	1
Enrofloxacin	0.125–16	1	>16	>16	>16
Erythromycin	0.5–8	8	>8	>8	>8
Florfenicol	0.5–16	≤ 0.5	4	1	2
Lincomycin	0.5–32	≤ 0.5	>32	≤ 0.5	2
Oxytetracycline	0.5–32	≤ 0.5	4	1	2
Spiramycin	0.5–16	≤ 0.5	>16	≤ 0.5	4
Tiamulin	0.0078125–16	0.0625	2	0.5	1
Tilmicosin	0.015625–32	0.0625	>32	1	>32
Tylosin	0.0078125–32	≤ 0.0078125	>32	0.0625	1

For each antibiotic, the dilution range, MIC minimum value detected, MIC maximum value detected, MIC50 and MIC90 are listed. All the values are expressed in μg/mL. MIC50 and MIC90 that were below or above the dilution range are marked with the sign “</>”.

Plates were incubated aerobically at 37±1°C and were checked within 24–48 hours after the inoculum, as soon as the positive-control-well showed typical signs of *Mycoplasma* growth. Each isolate was tested in duplicate. The MIC value was considered valid when both MIC tests produced identical results. Moreover, the MS reference strain (WVU 1853, NCTCC 10124) was added as a control strain in each work session.

The lowest antibiotic concentration that completely inhibited the growth/metabolism of the isolate *in vitro* after the incubation period was considered as the MIC value of the tested antimicrobials. When growth was not inhibited by the highest antimicrobial concentration, the MIC value was expressed as greater than (>) the highest antibiotic concentration present in the plate. On the contrary, if growth was inhibited by the lowest antibiotic concentration present in the plate, the MIC value was expressed as lower than or equal (≤) to this concentration.

Calculation of MIC50 and MIC90, namely the lowest concentration of an antimicrobial capable to inhibit 50% and 90% of bacterial isolates respectively, was performed.

### Statistical analyses

All statistical analyses were conducted under R environment (R^®^, R Foundation for Statistical Computing, Vienna, Austria, 2019). MIC class frequency variation along years was analyzed by Asymptotic Linear-by-Linear Association Test implemented in the package “coin” [38], considering the MS genotype as stratification variable. Tilmicosin MIC class frequency was in detail analyzed via a proportional odds model applying a logit link function and including the independent variables “year”, “genotype” and their interaction term. The “year” variable was coded as a numeric vector ranging from 0 to 6 by centering to 2012 the year of isolation. The proportional odds assumptions of the different independent variables—characteristic of this kind of model—was tested and proved correct. The proposed final model was constructed with the package “ordinal” (Ordinal: Regression Models for Ordinal Data. R package version 2019) and was obtained by a stepwise variable selection process based on sequential likelihood ratio tests (LR-tests) and elimination of variables with p-value > 0.05.

## Results

**Fig 1:**
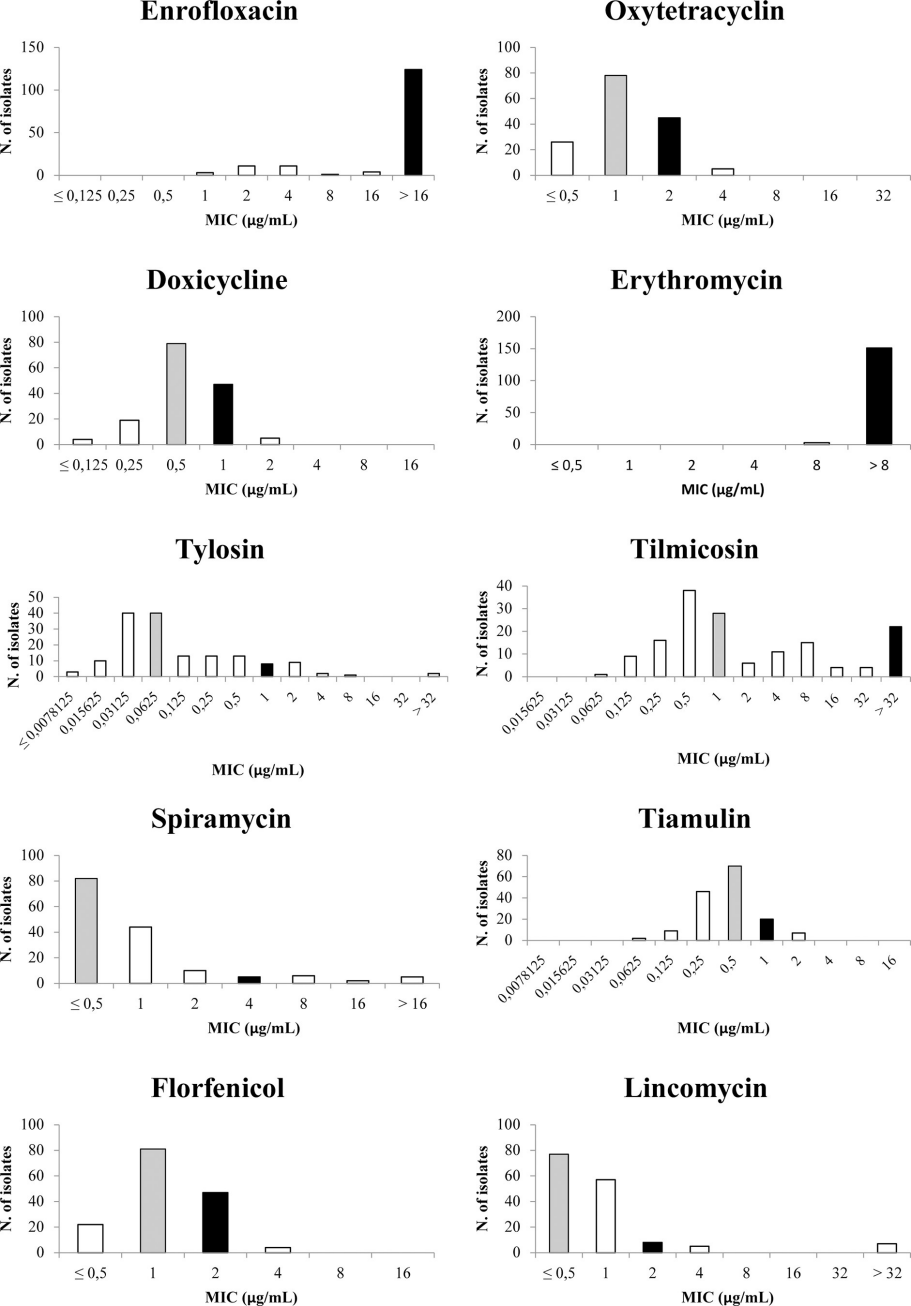
Minimum inhibitory concentration distribution (μg/mL) of enrofloxacin, oxytetracycline, doxycycline, erythromycin, tylosin, tilmicosin, spiramycin, tiamulin, florfenicol, and lincomycin. The number of isolates is present on the vertical axis of the ordinates and the antibiotic concentrations in μg per mL are displayed on the horizontal axis of the abscissas. Grey bars indicate the concentration that inhibits 50% of the isolates (MIC50 values). Black bars indicate the concentration that inhibits 90% of the isolates (MIC90 values). White bars indicate the other MIC values.

MIC results sorted by drug are shown in Table 2 and Fig 1. Because of the high number of isolates tested, all MIC values are schematically displayed in Table 3.

**Table 3:** The numerical consistency of MS isolates sorted by drug and drug concentration in the MIC plate.

Antibiotic	MIC values (μg/ML)														
	0.003906	0.0078125	0.015625	0.03125	0.0625	0.125	0.25	0.5	1	2	4	8	16	32	64
Oxytetracycline							26		78	45	5				
Doxycycline					4		19	79	47	5					
Enrofloxacin									3	11	11	1	4	124	
Erythromycin												3	151		
Tilmicosin					1	9	16	38	28	6	11	15	4	4	22
Tylosin	3		10	40	40	13	13	13	8	9	2	1			2
Spiramycin							82		44	10	5	6	2	5	
Tiamulin					2	9	46	70	20	7					
Lincomycin							77		57	8	5				7
Florfenicol							22		81	47	4				

The dilution range tested for each drug is indicated by the white cells in the table. Values located in the grey cells on the right indicate MIC values > the highest tested concentration. Values located in the grey cells on the left indicate MIC values ≤ the lowest tested concentration.

The results given by the type strain (WVU 1853, NCTC 10124) were consistent throughout the study indicating the good reproducibility of the test. All isolates showed concordance of the MIC results when tested in replicates.

Most of the 154 MS isolates (91%) showed MIC values of enrofloxacin greater than 2 μg/mL, 11 isolates (7.1%) showed MIC values of 2 μg/mL, and 3 isolates (1.9%) showed MIC values of 1 μg/mL. One hundred and twenty-four isolates (80.5%) had MIC values greater than the highest concentration of the antibiotic present in the plate (16 μg/mL). MIC50 and MIC90 values of enrofloxacin were the highest registered, together with those of erythromycin.

MIC values of tetracyclines were lower or equal to 4 μg/mL for all MS isolates. The MIC value patterns of oxytetracycline and doxycycline were very similar to each other, invariably characterized by just a one-dilution difference (see Fig 1). Growth was inhibited by the lowest concentration of oxytetracycline present in the plate (0.5 μg/mL) for 26 MS isolates (16.9%), whereas only 4 isolates (1.2%) were inhibited by the lowest concentration of doxycycline (0.125 μg/mL). A classical unimodal MIC value distribution was observed for both drugs, with 123 (79.9%) and 126 (81.8%) isolates for oxytetracycline and doxycycline respectively being located in the middle part of the graphic (Fig 1).

One hundred and fifty-one isolates (98%) resulted with MIC values greater than the highest concentration of erythromycin present in the plate (8 μg/mL), whereas only 3 isolates (2%) had a MIC equal to 8 μg/mL. An unimodal distribution of tylosin MIC values was noted, with 80 isolates (52%) showing MIC values between 0.03125 and 0.0625 μg/mL, 39 isolates (25.3%) showing MIC values between 0.125 and 0.5 μg/mL, and 17 isolates (11%) showing MIC values between 1 and 2 μg/mL. Only two isolates (1.3%) had MIC values of tylosin >32 μg/mL. These two isolates also had very high MIC values of the other tested drugs (see Table 4).

**Table 4:** Detailed list of the seven isolates showing a very high MIC value of lincomycin and their MIC values of all tested drugs.

ID	Year of isolation	*vlhA* Genotype	Poultry category	ENR	DOX	OXY	ERY	TYL	TILM	SPI	TIA	FL	LIN
IZSVE/2094	2013	F	B	>16	1	2	>8	0.5	>32	8	1	1	>32
IZSVE/5914	2013	F	B	>16	0.5	1	>8	>32	>32	16	0.5	1	>32
IZSVE/1067	2015	F	B	>16	0.25	1	>8	1	>32	8	0.5	1	>32
IZSVE/1116	2015	F	B	>16	1	2	>8	4	>32	>16	2	2	>32
IZSVE/2519	2015	F	T	>16	0.5	2	>8	8	>32	>16	2	2	>32
IZSVE/2518	2015	C	B	>16	1	2	>8	>32	>32	>16	2	4	>32
IZSVE/1925	2016	F	B	>16	0.5	1	>8	4	>32	>16	1	1	>32

ENR, enrofloxacin; DOX, doxycycline; OXY, oxytetracycline; ERY, erythromycin; TYL, tylosin; TILM, tilmicosin; SPI, spiramycin; TIA, tiamulin; FL, florfenicol; LIN, lincomycin; B, broiler; T, turkey.

Tilmicosin MIC values were characterized by a bimodal distribution in the graphic, with 66 isolates (42.9%) showing MIC values between 0.5 and 1 μg/mL, and 26 isolates (16.9%) showing MIC values ≥32 μg/mL. Most of isolates (83.1%) had MIC values ≤16 μg/mL.

With most of the isolates (81.8%) showing MIC values of spiramycin between ≤0.5 and 1 μg/mL, and only 13 isolates (8.4%) showing MIC values ≥8 μg/mL, spiramycin appeared to have an unimodal distribution in the graphic. The same data distribution was observed for tiamulin, since most isolates (95.5%) had MIC values equal or lower than 1 μg/mL. Only 7 isolates (4.5%) showed a MIC value of 2 μg/mL. With the exception of erythromycin, MIC50 values of macrolides were generally low or intermediate. Also, MIC50 and MIC90 of macrolides were located in distant points of the graphic (see Fig 1), while the opposite was observed for tiamulin and florfenicol, since MIC50 and MIC90 values differed for a one-fold dilution.

Florfenicol MIC values had an unimodal distribution, with half of the isolates (52.6%) showing a MIC value of 1 μg/mL. All isolates had MIC values ≤4 μg/mL.

Even though most isolates (87%) had MIC values of lincomycin between ≤0.5 and 1 μg/mL, 7 isolates (4.5%) showed a MIC value >32 μg/mL. These 7 isolates, all coming from meat poultry lineages, also showed high MIC values of the other tested drugs (see Table 4). The highest MIC values of lincomycin always corresponded to high MIC values of tilmicosin, but not vice versa.

The *vlhA* analysis revealed that 89 MS isolates out of 154 (57.8%) were D-type, 50 isolates (32.5%) were F-type, 14 isolates (9.1%) were C-type and 1 isolate (0.6%) was A-type (see Table 5). The numerical consistency of each genotype included in the study is coherent with the Italian prevalence during the study time.

**Table 5:** Number of isolates tested, year of isolation and *Mycoplasma synoviae vlh*A genotype.

Year of isolation	A	C	D	F	Total
2012	-	1	8	4	13
2013	1	2	9	13	25
2014	-	3	19	10	32
2015	-	3	23	9	35
2016	-	3	24	8	35
2017	-	2	6	6	14
2012–2017	1	14	89	50	154

**Fig 2:**
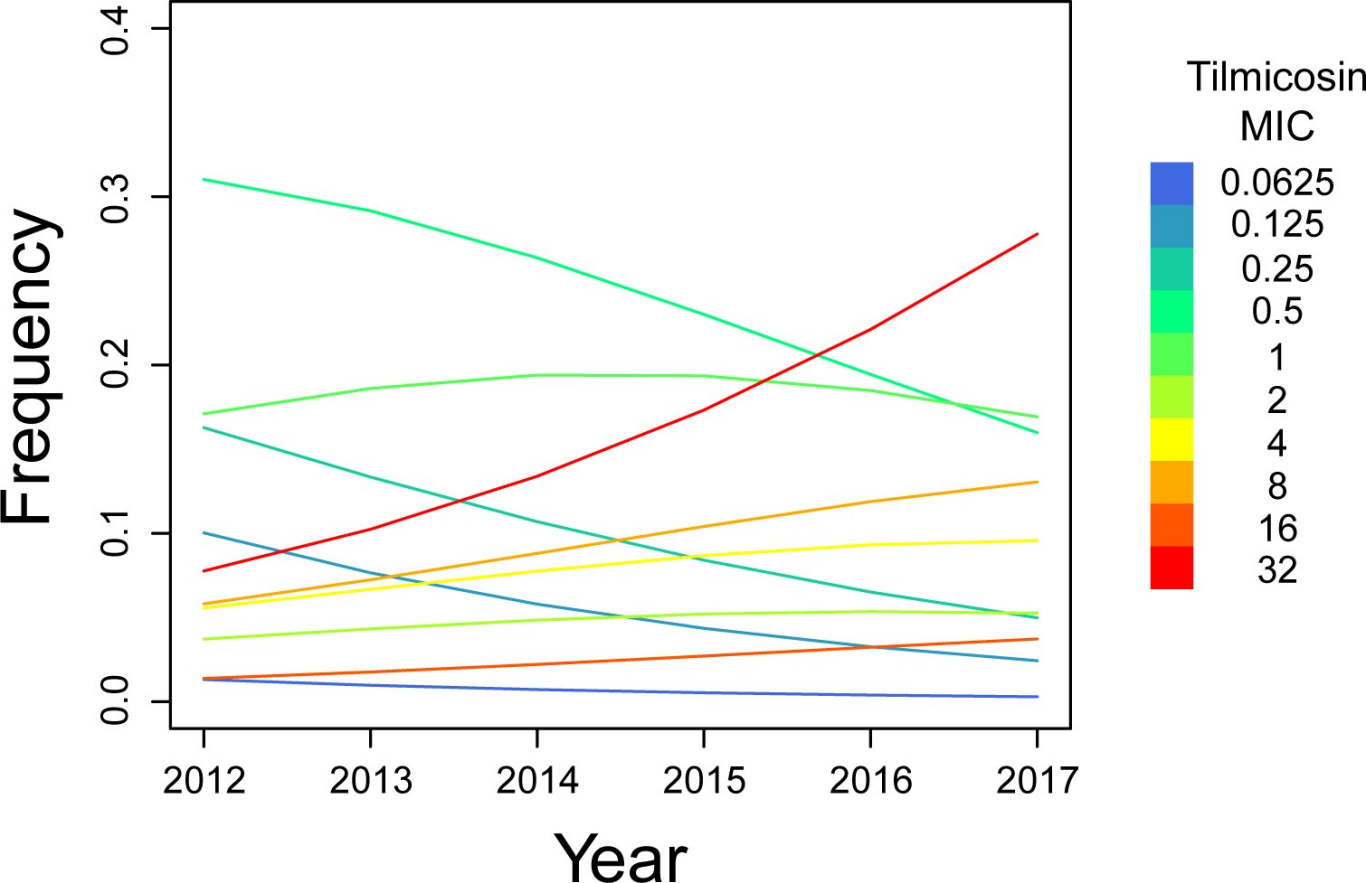
MIC classes frequency variations during the study time. The coloured bar indicates the different MIC classes. The lines in the graphic indicate the trend of the single classes throughout the years.

In order to determine whether there was a time-dependent variation in the frequency of observation of the different MIC values, we applied a linear-by-linear association test on all the tested antibiotics (see Table 6). The results showed that in the case of tylosin, tilmicosin, and tylvalosin—and with smaller significance, enrofloxacin and oxytetracycline—the distribution of the isolates among the different classes of MIC varied along the time of the study. In particular, in the case of tilmicosin, such variation seemed to suggest a steady, progressive increment in the frequency of isolates displaying high MIC values. To confirm this hypothesis, tilmicosin MIC data were analysed by means of a proportional odds model including the variable year of isolation (Year) (Table 7). According to the model, with one unit increase in year of isolation, the log of odds of observing increasing tilmicosin MICs stably increases by 0.304 (95% confidence interval: 0.117–0.494), with a p-value of 0.00154. Model predictions of MIC-frequency profile along the years 2012–2017 are showed in Fig 2 and clearly suggests a progressive drift towards high MIC values.

**Table 6:** Asymptotic Linear-by-Linear Association Test of antibiotic MIC-class frequencies versus years, stratified by genotype.

Antibiotic	Z-value	p-value
Doxicycline	-0.09	0.9283
Enrofloxacin	-2.22	0.02638
Florfenicol	0.10	0.92
Lyncomycin	1.20	0.2311
Oxytetracycline	1.98	0.04713
Spiramycin	1.00	0.3156
Tiamulin	-0.71	0.4799
Tylosin	3.82	0.0001324
Tilmicosin	3.68	0.0002314

**Table 7:** Proportional odds model parameter estimates with relative standard errors (Std.Error), Wald statistics (Z-value) and p-value.

	Estimate	Std. Error	Z-value	p-value
**Coefficients**				
Year	0.304	0.096	3.167	1.540 × 10^−3^
**Threshold coefficients**				
0.0625|0.125	-4.316	1.026	-4.205	2.610 × 10^−5^
0.125|0.25	-2.055	0.408	-5.032	4.854 × 10^−7^
0.25|0.5	-0.963	0.315	-3.056	2.243 × 10^−3^
0.5|1	0.350	0.286	1.220	0.222
1|2	1.139	0.304	3.751	1.761 × 10^−4^
2|4	1.354	0.313	4.327	1.511 × 10^−5^
4|8	1.738	0.328	5.299	1.164 × 10^−7^
8|16	2.296	0.350	6.560	5.381 × 10^−11^
16|32	2.475	0.358	6.912	4.779 × 10^−12^

## Discussion

Lately MS has been attracting more and more attention to the point it may be currently considered the most impactful *Mycoplasma* species in the poultry production system. This is mainly due to its wide diffusion worldwide [2], sometimes with a higher prevalence than MG, as it is in Italy. In the integrated poultry system, characterized by the presence of High Densely Populated Poultry Areas (HDPPA), mycoplasma infection spread is very eased. Mycoplasmas’ ability to be transmitted vertically makes chicken breeder groups responsible of generating several outbreaks located in distant areas, leaving the culling of the breeders as the only way for tackling the problem. On top of that, the high number of interconnections (feed truck, personnel, etc.) within the meat and the layer sectors represents the major critical point allowing the maintenance and the circulation of *Mycoplasma* strains. Choosing the effective antibiotic in case of MS infection could reduce the impact of the pathogen on several production parameters. At the same time, this would lead to an appropriate use of the antibiotics to which follows a reduction of their use in veterinary medicine. Even though antibiotics are licensed for specific use, therapeutic success is not always guaranteed; therefore, getting data on the susceptibility of currently circulating bacterial strains to antibiotics becomes crucial for the application of a proper antibiotic therapy. MIC is defined as the lowest concentration of an antibiotic capable of inhibiting the visible growth or metabolism of a microorganism *in vitro*. Knowing MIC values and keeping them updated allows standardizing the first-choice treatment.

Most of the previously published studies have been focusing on MIC values of few molecules for a small number of MS isolates. This is the first report on MIC values for a large number of MS isolates. Also, the chosen isolates come from one of the European HDPPA, and the most used antibiotics for MS treatment were tested. The data obtained indicate interesting differences compared to previous reports from other European and extra-European areas [16,18,22,23,25,27]. It must be said that the lack of standardized methods and media for MIC procedure for animal mycoplasmas makes results obtained by different laboratories hard to compare. In addition, no standardized or known breakpoints are available for any drugs, possibly resulting in different outcomes *in vitro* and *in vivo* [14,39]. Therefore, we preferred to describe MIC values obtained as low, intermediate or high instead of using the terms “sensitivity” or “resistance”.

We found that all MS isolates had high MIC values of enrofloxacin, long regarded as the drug of choice in case of *Mycoplasma* infection. The lowest MIC value of enrofloxacin detected in our study—given by only one isolate—was 2 μg/mL. Interestingly, this concentration corresponds to the highest MIC value of enrofloxacin obtained in previous reports. Dufour-Gesbert and collaborators [22] classified most (94.4%) of their MS isolates as intermediate with MIC values of 0.5–1 μg/mL. Two years later, Landman and collaborators [23] found that two out 17 isolates showed MIC values of enrofloxacin ≥2 μg/mL, although most isolates were classified as susceptible. In another study, the authors reported the presence in Israel of some MS isolates with MIC values of enrofloxacin above the resistance breakpoints (>1μg/mL) or within the intermediate range, even though most isolates were classified as susceptible [24]. In a recent study, Lysnyansky and collaborators [15] observed a decrease of susceptibility to enrofloxacin in 59% MS isolates coming from Israel and EU, Italy included. Also, they demonstrated that the presence of an amino acid substitution in the ParC protein is related to decreased susceptibility to enrofloxacin, with MIC values >1μg/mL. Reflecting upon the geographical distribution of the isolates with high MIC values of enrofloxacin, the authors concluded that a clonal spread might have taken place in a HDPPA. The high MIC values of enrofloxacin found in our study bring out the need of rehashing the use of some drugs in HDPPAs, especially when an initial shift towards high MIC values is noticed. To the authors’ opinion, if veterinarians had known about the resistance onset, they would have possibly chosen a different antimicrobial for MS infection treatment, reducing the selective pressure made by the use of enrofloxacin, and consequently avoiding the occurrence of high MIC values. This would have potentially let this molecule stay still in the list of the effective antibiotics against MS.

The tetracycline drugs we tested appeared to have a good performance since all MS isolates showed MIC values coherent to previous reports [22,23], even though with a slight increase. In particular, MIC values of tetracyclines showed a classical unimodal distribution. For most MS isolates, MIC values of doxycycline had a one-fold dilution difference compared to MIC values of oxytetracycline. This was confirmed by the MIC90 and MIC50 values comparison (see Fig 1). On the basis of the results obtained it may be proposed to test only one of the two molecules as the representative of the tetracycline class of antibiotics. Even though the isolates did not have very high MIC values of these two drugs, our results do not align with data from previous studies in Europe reporting MIC values of <0.06–0.125 μg/mL and <0.06–0.25 μg/mL for oxytetracycline and doxycycline respectively [22]. In 2008, Landman and collaborators [23] stated that all their isolates had a MIC <0.015 for doxycycline while we observed MIC values of <0.5–4 μg/mL for oxytetracycline and <0.125–2 μg/mL for doxycycline. This difference in the MIC value distributions of tetracyclines could be due to a different use in terms of quantity among countries, as suggested in another work [40], or to differences in antibiotic formulation. Indeed, MIC value ranges of tetracyclines for MS in Italy, the Netherlands and Argentina are 0.125–4 μg/mL, 0.015–0.25 μg/mL and 0.39-≥12.5 μg/mL respectively [21,23]. It is obvious that future studies on MIC will help understand MS sensitivity to tetracycline drugs and its possible decrease.

The MIC values of erythromycin we obtained confirm the intrinsic resistance of MS to this drug as previously suggested [26], and recently confirmed by Lysnyasky and collaborators [16] on the basis of a specific point mutation in position G2057A.

Despite the wide use of tylosin in poultry production, only two isolates—collected in 2013 and in 2015 from broiler chickens—showed a very high MIC value of tylosin (>32 μg/mL). These two isolates also showed very high MIC values of the other drugs (see Table 5), especially of lincomycin (>32 μg/mL) and tilmicosin (>32 μg/mL). However, isolates with high MIC values of tylosin were not found during the following years (2016 and 2017), leaving the question open as to why they did not spread in the poultry population, a quite controversial occurrence. The development of resistance to tylosin for MS *in vitro* has been demonstrated to be faster when compared to enrofloxacin [26,28]. Keeping that in mind, and suggesting that tylosin is commonly used in poultry, the number of MS isolates with high MIC values should have had an increase over the years, but this was not the case. It is known that it is not the exposure to antimicrobials itself which is responsible for the reduction of sensitivity to drugs. In fact, a mutation or a specific genetic combination allows the bacteria to survive in the presence of certain concentrations of antimicrobials [41,42]. Regarding to MS, a significant decrease in susceptibility to tylosin, tilmicosin, and lincomycin related to mutations A2058G or A2059G in V domain of one or both 23S rRNA alleles was reported [16]. One of the hypotheses is that, although a certain decrease in susceptibility occurred, something hindered the spread of the “resistant” strains within the animal population, in contrast to what was proposed for enrofloxacin [15]. A possible explanation of this phenomenon could be found in the consequences linked to the acquisition of a mutation by the isolates in question. An example of this eventuality is *in vitro*-selected *Helicobacter pylori* mutants (2058U) that showed higher resistance to antibiotics but strongly decreased growth. Also, it was observed that this mutation would be readily lost in absence of the selective pressure made by the antibiotic [43,44]. It is possible that mutations can weaken the bacterial strain but, on the other hand, would let it be advantaged in surviving during the antibiotic treatment unlike the wild type. However, once the antibiotic treatment is over, the wild strain could grow back bringing about the disappearance of the mutated strain. It is obvious that further studies are needed to thoroughly understand this occurrence, which might be also related to the *Mycoplasma* species itself. As an example, more than half of the *M*. *bovis* isolates (61%) examined by Barberio and collaborators [40] showed MIC values of tylosin >32 μg/mL. Moreover, Klein and collaborators [39] observed notable behaviour differences in *M*. *bovis* and *M*. *hyopneumoniae* against tylosin, with MIC_50_/MIC_90_ values being 32/>64 μg/mL and 0.03/0.125 μg/mL respectively.

The MIC values distributions of tilmicosin and spiramycin were slightly different to that of tylosin. Twenty-six isolates (16.9%), including the two isolates with MIC value of tylosin >32 μg/mL, had MIC values of tilmicosin ≥ 32 μg/mL. However, high MIC values of tilmicosin did not always correlate with high MIC values of tylosin (see Table 4). Indeed, six isolates had MIC values of tylosin 0.5 μg/mL, which is considered to be a low MIC value. In general, most drugs (tylosin, tilmicosin, enrofloxacin and oxytetracycline) showed significant fluctuations in MIC frequencies along the time of observation. However, in the case of tilmicosin, we observed a clear and statistically significant drop in the frequency of isolates with low MIC values counterbalanced by an increase in frequency of high MIC isolates. This observation seems to suggest a progressive drift towards high MIC values and contrasts with a recent study showing that 90% of strains from Eastern Europe displayed low MIC values (≤1 μg/mL) [25]. Further analyses are needed to investigate the molecular mechanism responsible of the observed resistance. That said, in our opinion it would not be possible to assess which macrolide drug could be the representative of this class of antibiotics, at least for MS. As a consequence, there is the need of testing both molecules in future studies, possibly widening the drug concentration ranges, in order to confirm our findings and/or better understand the behaviour of MS against macrolides. Interestingly, the MS isolates showing high MIC values of tilmicosin were found in the turkey or the broiler sectors but not in the layer one. A possible explanation for the absence of this kind of isolates in the layer sector could be the withdrawal time for tilmicosin when administered to laying hens.

Even though most of the MS isolates had low MIC values of spiramycin, 7 isolates (4.5%) showed MIC values of spiramycin ≥16 μg/mL and high MIC values of tilmicosin and lincomycin. It is worth noting that six of these isolates were collected from the broiler sector, the only poultry category spiramycin is licensed for in Italy.

We tested tiamulin, the only drug of the pleuromutilins antimicrobial class currently approved in Italy in different poultry categories, even though it is used mainly in the layer sector because of a toxic effect when administered together with some anticoccidian drugs [45]. Our data reveal an unimodal distribution with most isolates located in the middle of the graphic (Fig 1), between 0.25 and 1 μg/mL. However, high MIC values of tiamulin corresponded to high MIC values of other drugs (see Table 4).

Florfenicol was included in this study because it could be a good candidate for the treatment of avian mycoplasmosis in the future, although this drug is only licensed for pigs in Italy since it is considered effective against *Mycoplasma hyopneumoniae* [46]. Even though florfenicol is not used in poultry, the MIC values distribution reported in our study is characterized by most part of isolates being allocated between 1 and 2 μg/mL. We noted that the MIC values of florfenicol fall in a narrow range, but specific breakpoints for MS are needed to better establish the effectiveness of this molecule. MS isolates showing the highest MIC values of florfenicol also showed high MIC values of other drugs, including tylosin, tilmicosin and lincomycin.

Regarding lincomycin, the MIC value distribution showed two main peaks, the first one located at <0.5 μg/mL and at 1 μg/mL, while 7 isolates (4.5%) showed the highest MIC value (>32 μg/mL). Interestingly, all of these seven isolates had the highest MIC value of tilmicosin as well, but not vice versa. It is reported that resistance to the lincosamides can develop alone but cross-resistance between macrolides and lincosamides occurs more commonly [47]. This cross-resistance could be “constitutive resistance” or “dissociated inducible cross-resistance”. In the first case, bacteria show high MIC values to both drugs, while in the second one bacteria are initially resistant to macrolides but totally susceptible to lincosamides and, in a second time, they can develop resistance to lincosamides when exposed to macrolides. This might be the case of the MS isolates with high MIC values of tilmicosin but low MIC values of lincomycin, however we did not investigate the presence of this resistance mechanism.

In conclusion, knowing MIC values for this impactful *Mycoplasma* could eventually allow designing drug rotation plans with the ultimate aim of avoiding a decrease in the susceptibility to antimicrobials, as is already the case for anticoccidial drug use in poultry. It will be fundamental to keep studying MIC value fluctuations during the time in order to create a significant database, covering both micro- and macro-areas. This collection of data would support veterinarians in selecting the proper drug for treating animals, aiming at the conscious use of antibiotics with consequential preservation of their efficacy and the reduction of their use in veterinary medicine as inspired by the “One-Health” approach.

## Supporting information

S1 Table *Mycoplasma synoviae* isolates list. The name of the single isolates selected for the study is reported in the table together with the genotype, the poultry sector from which they were isolated, and the MIC data sorted by drug.
(XLSX)
